# Phytochemical-Mediated Modulation of Doramectin Pharmacology in Sheep: Exploring the Cinnamaldehyde–Pink Grapefruit Combination

**DOI:** 10.3390/ani15172539

**Published:** 2025-08-29

**Authors:** María Victoria Miró, Paula Ichinose, Mercedes Lloberas, Lucila Moriones, Carlos Lanusse, Guillermo Virkel, Adrián Lifschitz

**Affiliations:** 1 Laboratorio de Farmacología, Centro de Investigación Veterinaria de Tandil (CIVETAN), UNCPBA-CICPBA-CONICET, Campus Universitario, Tandil B7000, Buenos Aires, Argentina; vmiro@vet.unicen.edu.ar (M.V.M.); paulaichinose@vet.unicen.edu.ar (P.I.); lucilamoriones@vet.unicen.edu.ar (L.M.); clanusse@vet.unicen.edu.ar (C.L.); gvirkel@vet.unicen.edu.ar (G.V.); 2 Facultad de Ciencias Veterinarias, Universidad Nacional del Centro de la Provincia de Buenos Aires (UNCPBA), Campus Universitario, Tandil B7000, Buenos Aires, Argentina; 3 Laboratorio de Parasitología, Instituto Nacional de Tecnología Agropecuaria (INTA), Estación Experimental, Balcarce B7620, Buenos Aires, Argentina; lloberas.maria@inta.gob.ar

**Keywords:** cinnamaldehyde, pink grapefruit, doramectin, P-glycoprotein, anthelmintic resistance

## Abstract

Phytotherapy represents a promising field for developing new therapeutic strategies, either by acting as antiparasitic agents themselves or by enhancing the efficacy of synthetic drugs. The present study evaluated the in vivo pharmacokinetic and pharmacodynamic interactions following the administration of doramectin, either alone or in combination with cinnamaldehyde and pink grapefruit essential oil (CNM–PGF). Additionally, ex vivo P-glycoprotein-mediated interactions were assessed at the intestinal level. Higher efficacy against *Haemonchus contortus* was observed with the combined treatment of doramectin plus phytochemicals or loperamide. Although phytochemicals and loperamide reduced the ex vivo efflux of rhodamine 123, no pharmacokinetic interactions were detected in vivo. The enhanced efficacy is most likely attributable to localized drug–drug interactions and increased drug availability at the parasite interface. Based on the results obtained, the best treatment would be the combination of DRM with phytochemicals such as CNM–PGF and loperamide. However, a pharmaceutical formulation that allows sustained in vivo interaction between these compounds is needed.

## Simple Summary

In the context of increasing anthelmintic resistance, the search for novel strategies to control gastrointestinal nematodes in ruminants is urgent. Various plant-derived products have been evaluated—primarily through in vitro studies—as potential alternatives or complements to conventional anthelmintic treatments. Notably, phytochemicals may serve as valuable adjuncts by interacting with synthetic anthelmintics and potentially enhancing their efficacy. Furthermore, it is crucial to investigate whether these compounds can modulate resistance mechanisms, particularly those mediated by transporter proteins such as P-glycoprotein. The present work reports an integrated pharmaco-parasitological study designed to evaluate the combined use of doramectin with cinnamaldehyde and pink grapefruit essential oil. In addition, ex vivo experiments were conducted to explore P-glycoprotein interactions at the intestinal level. The development of new pharmacological tools remains a critical challenge in advancing sustainable parasite control strategies.

## 1. Introduction

Gastrointestinal nematode infections are a major cause of economic losses in ruminant production systems globally [1]. Control of nematodes has long relied on the use of broad-spectrum anthelmintics, particularly macrocyclic lactones (MLs). However, the overuse and misuse of these drugs have led to the emergence and spread of resistant nematode populations [2,3]. Resistance to MLs is complex and multifactorial, involving changes in drug targets and the overexpression of efflux transporters such as P-glycoproteins (P-gps) [4,5]. P-gps are ATP-binding cassette (ABC) transporters located in host epithelial barriers and in different tissues of nematodes, where they play a role in limiting drug accumulation and facilitating detoxification [6,7]. Their involvement in ML resistance has been demonstrated in both field and laboratory studies [8,9], highlighting the potential of P-gp inhibitors as a pharmacological tool to restore anthelmintic efficacy [10,11,12].

Phytotherapy represents a promising field for exploring new therapeutic options, with the challenge of the therapies acting as antiparasitic agents themselves or enhancing the efficacy of synthetic drugs. In this context, natural compounds such as monoterpenes have the potential to serve as sources of novel therapeutic agents [13]. Phytochemicals are now being evaluated as possible parasite control methods in livestock [14,15,16]. Phytochemicals can be incorporated into feed as encapsulated essential oils [15,17] or administered via oral drenches, such as emulsions. Some attempts have been made to develop nanoemulsions, which were evaluated in vitro against *Haemonchus contortus* [18]. In addition to its own anthelmintic activity, phytochemicals may be combined with synthetic anthelmintics to enhance their anthelmintic action. As a result of pharmacokinetic and/or pharmacodynamic interactions, bioactive phytochemicals may increase the efficacy of existing anthelmintic drugs. Among natural compounds with pharmacological properties that can modulate P-gp activity, cinnamaldehyde (CNM)—a phenylpropanoid derived from cinnamon—and limonene present in pink grapefruit (PGF) have attracted attention. CNM has been shown to inhibit the P-gp-mediated transmembrane transport of puerarin from the basolateral to the apical side, which may be relevant for its therapeutic use in ischemic stroke [19]. The grapefruit juice components responsible for clinically relevant drug interactions through modulation of P-gp are primarily the flavonoid naringin and the furanocoumarin bergamottin [20]. However, grapefruit essential oil contains these compounds only in low proportions. Limonene, a major constituent of various citrus essential oils, has also demonstrated interactions with P-gp in *H. contortus* [21,22]. Given the well-established interaction between P-gp and MLs [6,23], along with the confirmed impact of P-gp modulators such as loperamide (LPM) on ML efficacy in lambs [11], there is a strong mechanistic basis to investigate the potential of natural products like CNM and limonene to alter the pharmacokinetics and antiparasitic activity of MLs.

Given this context, the present study aimed to conduct a comprehensive pharmacological evaluation of doramectin (DRM), its combination with CNM and PGF, and the coadministration of DRM plus LPM in lambs naturally infected with ML-resistant nematodes. The specific objectives were (i) to assess in vivo anthelmintic efficacy through fecal egg count reduction tests (FECRT), (ii) to evaluate the influence of CNM–PGF and LPM on P-gp-mediated intestinal transport of DRM, and (iii) to characterize potential pharmacokinetic interactions affecting drug absorption and systemic exposure.

## 2. Materials and Methods

### 2.1. Evaluation of the Efficacy of the Coadministration of DRM and CNM–PGF

The coadministration of DRM and CNM–PGF, both by an oral emulsion, was evaluated in lambs. The trial involved 35 Corriedale and Texel crossbred lambs (mean body weight, 26 kg) naturally infected with resistant gastrointestinal nematodes and was conducted in a sheep experimental unit (Estación Experimental INTA, Balcarce, Argentina). There, a parasite control program based on the intensive use of antiparasitic drugs has been implemented for many years, leading to anthelmintic resistance to MLs and benzimidazoles. Animals were selected based on worm egg per gram counts (EPG), with an average EPG count of 2102 ± 1662. Animals were kept in a paddock and fed hay ad libitum, together with commercial concentrate feed. All animals had free access to water. Lambs were assigned to four experimental groups sorted by EPG count. Group A received an emulsion containing DRM (single dose of 0.2 mg/kg) (*n =* 9). Group B received CNM–PGF orally as an emulsion (two doses of 100 mg/kg every 24 h) (*n* = 9). Lambs of Group C were treated with an emulsion containing both DRM and CNM–PGF on the first day, and the second day, they received an emulsion of CNM–PGF only (*n* = 9). Group D included untreated lambs (*n* = 8). The selected phytochemical dose was based on previous in vivo studies with other monoterpenes, such as thymol and carvone, which were administered to lambs at doses ranging from 100 to 150 mg/kg, without adverse effects [16,17]. CNM–PGF was formulated as an emulsion composed of Tween-80/sesame oil (3:1) 20% at a final concentration of 15% CNM and 15% PGF. Briefly, the CNM–PGF emulsion was prepared using the high-energy ultrasonic method. CNM and PGF were mixed with sesame oil to form the oil phase. The oil phase was added dropwise to the aqueous phase containing Tween-80 and distillated water while stirring, and the emulsion formed was then subjected to ultrasonic emulsification for 15 min. The same steps were performed for the DRM + CNM–PGF emulsion, the synthetic compound was added to the oil phase, and their final concentration was 0.03%. Throughout the course of 14 days, visual inspection was used to confirm that the emulsions remained stable at room temperature and to look for signs of creaming or breaking. To evaluate the effect of the treatment, individual fecal samples from all the lambs were collected on days −1, 7, and 14 of treatment, and the fecal egg count reduction (FECR) was calculated. The coprocultures were prepared with 10 g of feces from a pool of each experimental group obtained on days −1 and 14. The nematode genera and species were identified through the third-stage larvae recovered from the coprocultures [24].

### 2.2. Ex Vivo Assessment of Intestinal Transport-Mediated Interactions

A series of ex vivo experiments were performed to evaluate the interaction of DRM, CNM–PGF, or LPM with ABC transporters. The modulation of intestinal transport of rhodamine 123 (Rho123) was assessed using the diffusion chamber model. For this assay, sheep ileum samples were obtained from a local slaughterhouse (Ayacucho, Argentina). Ileum samples were obtained from Corriedale and Texel crossbreed lambs of approximately 31 kg in weight; immediately after extraction, samples were rinsed gently with ice-cold KCl 1.15% and conserved in Euro-Collins solution (0.19 M glucose; 15.43 mM KH_2_PO_4_; 42.48 mM K_2_HPO_4_; 15.02 mM KCl; 10 mM CO_3_HNa) at 4 °C during transport to laboratory facilities. The incubation process was started immediately after obtaining the tissue. During the preparation and assembly process, intestinal tissue was always maintained between 0–4 °C. The tissue was then mounted vertically in a diffusion chamber with an internal surface area of 0.8 cm^2^. The bathing solution consisted of Krebs buffer (1 mM NaH_2_PO_4_-H_2_O; 2.5 mM Cl_2_Ca-2H_2_O; 4.7 mM KCl; 1.2 mM Cl_2_Mg-6H_2_O; 0.004 mM EDTA; 11.1 mM glucose; 118 mM NaCl, 25 mM Na_2_CO_3_; 0.11 mM ascorbic acid). Both the mucosal (M) and serosal (S) sides were filled with 7 and 5 mL of prewarmed and oxygenated Krebs buffer, respectively. To ensure oxygenation, chambers were incubated in an orbital shaker (Ferca, Buenos Aires, Argentina) set at 60 rpm and maintained at 37 °C under a humidified atmosphere of 95% O_2_:5% CO_2_. During preparation, chambers were preincubated to slough off any dead cells, and during this period, the tissue were exposed in the donor side to the presence or absence of 5 µM of DRM; 1.5 mM of CNM–PGF; or 50 µM of LPM, a well-established P-gp modulator, which was used as a positive control [25,26]. After a 20 min equilibration period, Krebs buffer was completely replaced with fresh medium fortified with 0.5 µM of Rho123 as a substrate to the M or S side of the chambers for measuring the absorption and secretion processes, respectively. Thus, Rho123 was incubated alone (control assays) or in the presence of DRM, CNM–PGF, or LPM. Tissues were incubated for 4 h, and each hour, aliquots of 1 mL were collected from the receptor side and replaced with fresh Krebs buffer.

### 2.3. In Vivo Pharmacokinetic/Pharmacodynamic Assessment of DRM Modulation

An in vivo assay was performed to evaluate the pharmacokinetics and the efficacy of DRM orally administered alone or in combination with CNM–PGF and LPM against resistant gastrointestinal nematodes in lambs. For the trial, 27 naturally infected Corriedale and Texel crossbreed lambs (average weight, 33 kg) were used. On day −1, all lambs were checked for EPG and tagged, and the individual body weights were recorded. As in the previous assay (Trial 2.1), the selection of the animals was based on EPG. Selected animals displayed an average EPG count of 1504 ± 745 eggs. Lambs were housed in a paddock with access to hay, commercial concentrates, and water ad libitum. Lambs were assigned into three experimental groups. Group A received DRM (0.2 mg/kg) as an oral emulsion (*n* = 7). Lambs in groups B and C received an emulsion containing DRM (a single dose of 0.2 mg/kg) and either LPM (*n* = 7) (three oral doses of 0.3 mg/kg, every 24 h) or CNM–PGF (*n* = 7) (three oral doses containing 72 and 64 mg/kg of CNM and PGF, respectively, every 24 h). Group D served as the untreated control (*n* = 6). As appropriate, the final concentrations of the active ingredients within the formulations were DRM 5%, CNM 18%, PGF 16%, LPM 30%. The compounds were diluted in Tween-80 11%, sesame oil 1–30%, and distilled water. The formulations were prepared using a similar method to that used in the previously described trial (Trial 2.1).

For the plasma disposition of DRM, jugular blood samples (2 mL) were collected into heparinized vacutainer tubes before treatment and at 3, 6, 9, 24, 30, 48, and 54 h and 3, 4, 7, 10, and 14 days post treatment. Blood samples were centrifuged at 2000× *g* for 15 min; the recovered plasma was kept in labeled vials and stored at −20 °C until the analysis of DRM by high performance liquid chromatography (HPLC). The effect of each treatment was indirectly estimated by collecting fecal samples on days −1 and 14 of administration to evaluate the FECR.

### 2.4. Analytical Procedures

#### 2.4.1. Chromatographic Analysis

DRM concentrations in plasma were measured by HPLC with fluorescence detection according to the methods described by reference [27] and modified by reference [28]. Briefly, a 0.25 mL aliquot of the plasma sample was combined with 10 ng of the internal standard compound (moxidectin) and then mixed with 0.75 mL of acetonitrile. The sample mixture was centrifuged at 2000× *g* for 10 min at 4 °C, and the supernatant was manually transferred into a tube and concentrated to dryness under a stream of nitrogen. The derivatization of DRM was conducted following the technique described by reference [29]. DRM concentrations in plasma were determined by HPLC using a Shimadzu 10 A HPLC system with fluorescence detection reading at 365 nm (excitation) and 475 nm (emission wavelength). Calibration curves were prepared in the range between 0.25 and 200 ng/mL. Correlation coefficients (r) and coefficients of variation (CV) were calculated. The linear regression lines for DRM showed correlation coefficients ≥ 0.99.

#### 2.4.2. Intestinal Efflux Analysis

Samples from the receptor side (1 mL) were mixed with 2 mL of distillated water to reach a final volume of 3 mL. The concentrations of Rho123 were measured using a fluorescent spectrophotometer RF-5301PC (Shimadzu Corporation, Kyoto, Japan) set at excitation and emission wavelengths of 485 and 520 nm, respectively [30]. The calibration curve was achieved in a range between 0.008 and 35 ng/mL.

Values of unidirectional transepithelial effective permeability (*P_eff_*) (cm.s^−1^) were calculated for each chamber according to the following equation:

$$P_{eff}=\left(\frac{dC}{dT}\right)\left(\frac{1}{\left(A.C_{0}\right)}\right)$$

The appearance rate on the receiving compartment is *dC*/*dT*, calculated from the slope of the concentration versus time curve over a time period of 4 h (Rho123); A is the exposed area (cm^2^) of the tissue in the Ussing chamber (0.8 cm^2^), and *C*_0_ is the initial drug concentration (ng/mL) in the donor compartment. The efflux ratio (*ER*) was calculated as follows:

$$ER=\frac{mean\;P_{eff}\;S-M}{mean\;P_{eff}\;M-S}$$

The *P_eff_* reflects the ability of one compound to permeate a cell layer. The intestinal absorption is represented by permeability in the *M*–*S* direction. The *S*–*M* side can also be used to measure permeability, in this case characterizes intestinal secretion. A higher *P_eff_ S*–*M* compared to the *P_eff_ M*–*S* is indicative of carrier-mediated transport. Thus, substrates of efflux transporters expressed on the apical surface (as Rho123) are transported more rapidly in the *S*–*M* direction, and their *ERs* are >2 [31].

### 2.5. Data Analysis

Data are expressed as mean ± standard deviation (SD). Rho123 transport across the ileum is presented as ng of Rho123 in the receptor side of the chamber. The plasma concentration versus time curves obtained after treatment of each animal were fitted with the PK Solutions 2.0 software (summit Research Services, Ashland, OH, USA) software. Pharmacokinetic parameters were determined using a noncompartmental model method [32]. The evaluation of the *FECR* was calculated according to the following formula [33], with modifications:

$$FECR\;\left(\%\right)=100\;\times \;(1-\frac{T2}{T1})$$

where *T*1 and *T*2 are the arithmetic mean EPG counts in the treated group on days 0 and 14, respectively. The 90% confidence intervals were calculated following the method of reference [34].

The statistical analysis was performed using Prism 8.0 (GraphPad Software, San Diego, CA, USA). ANOVA or Kruskal–Wallis tests were used for the statistical comparison. Tukey or Dunn tests were used for post hoc multiple comparisons. The egg counts obtained for each treatment on Day 14 were compared with the corresponding baseline counts on Day 0 using the Wilcoxon matched-pairs signed rank test. Differences were considered statistically significant at *p* < 0.05.

## 3. Results

### 3.1. Evaluation of the Efficacy of the Coadministration of DRM and CNM–PGF

The results of the FECRT and the efficacy by nematodes genus are shown in Table 1 and Table 2, respectively. The initial mean EPG values in the experimental animals were 2102 ± 1662. There was no significant change in the EPG count in the control group between day 0 and day 14 post-treatment. The predominant parasitic genera identified in prior treatments were *Haemonchus* spp. (48%), *Ostertagia* spp. (38%), *Trichostrongylus* spp. (11%), and *Cooperia* spp. (9%). No significant differences were observed in the mean EPG counts on day 14 post-treatment after the administration of CNM–PGF alone (1615 ± 1129) compared to DRM alone (910 ± 2548), whereas the combined DRM + CNM–PGF (529 ± 1587) treatment showed a significant reduction compared to that observed after the administration of phytochemicals alone. The prevalence of egg count negativization on day 14 (i.e., the percentage of animals with an egg count of zero) was 0%, 40%, and 89% following treatment with CNM–PGF, DRM, and DRM + CNM–PGF, respectively. Notably, the combined DRM + CNM–PGF treatment increased the efficacy against resistant *H. contortus* from 0% to 79%.

### 3.2. Ex Vivo Assessment of Intestinal Transport-Mediated Interactions

**Figure 1:**
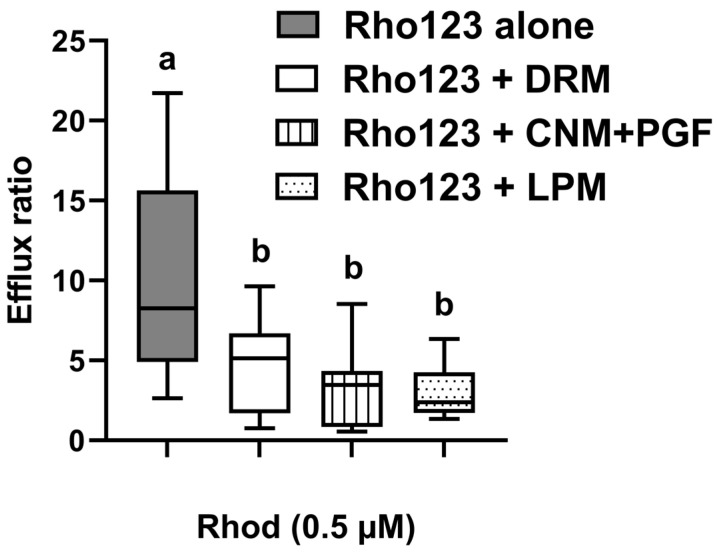
Efflux ratio (*P_eff_* S–M/*P_eff_* M-S) of rhodamine 123 (Rho123) across sheep ileum following its incubation either alone or in the presence of doramectin (DRM), cinnamaldehyde plus pink grapefruit essential oil (CNM–PGF), and loperamide (LPM). Values are expressed as median (min–max) (*n* = 8–11 determinations). Different letters indicate significant differences (*p* 0.0078).

The validation for the Rho123 detection by fluorometry demonstrated a linear regression line with a correlation coefficient of ≥0.9999, indicating strong analytical performance. The coefficient of variation ranged between 1.47 and 5.79%, confirming acceptable repeatability. The ex vivo diffusion study revealed asymmetric transport of Rho123 across the sheep ileum. Rho123 absorption expressed as *P_eff_* M–S was 1.39 × 10^−6^ ± 3.43 × 10^−7^ cm/s, whereas the secretion of Rho123 (*P_eff_* S–M) was significantly higher (1.11 × 10^−5^ ± 3.64 × 10^−6^ cm/s), indicating active efflux. The presence of DRM, CNM + PGF, and LPM led to increased intestinal absorption of Rho123. Specifically, the *P_eff_* M–S values were 3.83 × 10^−6^ ± 1.87 × 10^−6^ cm/s for DRM (*p* = 0.2337), 7.24 × 10^−6^ ± 1.81 × 10^−6^ cm/s for CNM–PGF (*p* = 0.0093), and 3.84 × 10^−6^ ± 1.02 × 10^−6^ cm/s for LPM (*p* = 0.0497). Conversely, the presence of these modulators did not produce significant changes in the S to M transport of Rho123, with *P_eff_* S–M values ranging between 1.00 × 10^−5^ cm/s and 2.80 × 10^−5^ cm/s (*p* > 0.05). In fact, all modulators significantly reduced the ER of Rho123 by approximately 60–70%, indicating inhibition of transporter-mediated efflux. The comparative ER under each condition is presented in Figure 1.

### 3.3. In Vivo Pharmacokinetic/Pharmacodynamic Assessment of DRM Modulation

**Figure 2:**
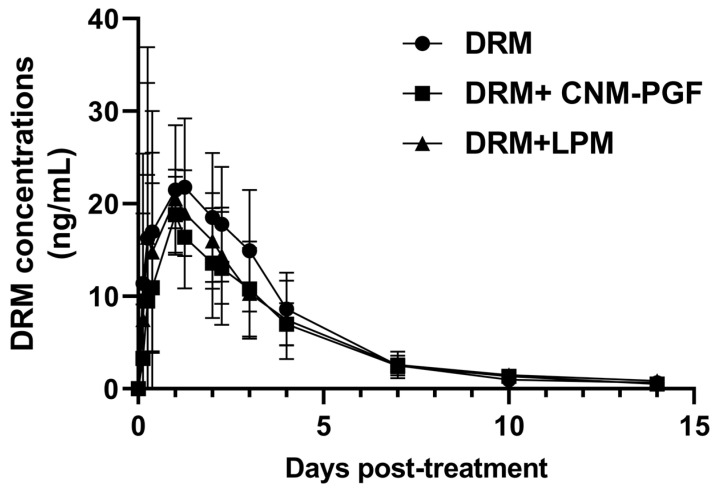
Comparative plasma concentration profiles obtained after the oral administration of doramectin (DRM), alone or combined with cinnamaldehyde plus pink grapefruit essential oil (CNM–PGF) or loperamide (LPM), to lambs (*n* = 7). Values are expressed as mean ± SD.

DRM was detected in the plasma of treated sheep from 3 h to 14 days post-administration across all three experimental groups. No significant differences were observed in the plasma disposition profiles following the administration of DRM alone or in combination with CNM–PGF extract or LPM. The plasma concentration profiles of DRM are shown in Figure 2. Key kinetic parameters for DRM reflecting systemic availability (Cmax and AUC) and its elimination half-life were not significantly altered by the co-administration with CNM–PGF or LPM. These pharmacokinetic parameters are summarized in Table 3.

The initial mean EPG count in the experimental sheep was 1504 ± 745. No significant change in the EPG was observed in the control group between day 0 and day 14 post-treatment. The predominant parasitic genera identified before treatments were *Haemonchus* spp. (10%), *Ostertagia* spp. (25%), *Trichostrongylus* spp. (55%), and *Oesophagostomum* spp. (10%). On day 14 post-treatment, no significant differences in mean EPG counts were observed after the administration of DRM alone (694 ± 944) compared to the results for DRM + CNM–PGF (762 ± 1565) and DRM + LPM (397 ± 520). A similar prevalence of egg count negativization (29%) on day 14 was observed across all groups. However, genus-specific analysis revealed that the combined treatments of DRM + CNM–PGF and DRM + LPM improved efficacy against *Haemonchus* spp., increasing the percentage of reduction from 0% (DRM alone) to 29% and 79%, respectively. The results of the FECRT and the efficacy by nematodes genus are shown in Table 4 and Table 5, respectively.

## 4. Discussion

The emergence and spread of anthelmintic resistance among gastrointestinal nematodes pose a serious threat to sustainable small ruminant production worldwide. MLs have been used as cornerstone drugs for parasite control for decades, but increasing reports of resistance, particularly among *H. contortus*, have compromised their efficacy [8]. Given the potential of novel therapeutic approaches, it is essential to ensure the safety of the compounds employed. Both CNM and limonene are considered to have low toxicity profiles. Limonene, the main constituent of PGF essential oil, is regarded as practically non-toxic, with an oral LD_50_ in rats exceeding 5000 mg/kg [35]. Similarly, CNM shows low toxicity, with an oral LD_50_ in rats of approximately 2220 mg/kg [36]. Previous studies have evaluated the efficacy of essential oils or single phytochemicals such as thymol and carvone against gastrointestinal nematodes in sheep, reporting variable results ranging from low efficacy to promising outcomes [16,17,37,38,39]. Whereas administration of single phytochemicals such as thymol and carvone resulted in low to moderate (0–47%) efficacy [16,17], treatment with an essential oil rich in p-cymene and carvacrol achieved 68% efficacy against mixed natural infections in lambs [39]. Similarly, supplementation of fattening lambs with a feed additive containing carvacrol and limonene reduced nematode egg counts by approximately 59% [38]. Based on previous evidence of synergistic in vitro activity between phytochemical combinations [40], we selected the CNM–PGF combination for evaluation in the present trial. The CNM–PGF combination demonstrated a modest overall reduction in fecal egg counts (17%). However, genus-specific analysis revealed a higher efficacy against *H. contortus*, with a 37% reduction in egg output (Table 2). CNM has previously been evaluated for its nematocidal activity. Different effects of cinnamaldehyde (CNM) on nematodes have been reported [41], demonstrating that CNM may serve as a promising natural alternative to synthetic antiparasitic drugs for nematode control. Exposure to CNM significantly altered the expression of metabolic genes in *C. elegans*, particularly those involved in glutathione metabolism. Additionally, the potential antiparasitic mechanism of CNM has been recently described [42], showing that CNM reduces acetylcholine and GABA currents and decreases both channel activity and open duration in native muscle of *C. elegans*, indicating an inhibitory effect. Although it exhibited in vitro efficacy against *C. elegans*, no in vivo efficacy was observed in rats infected with *Syphacia muris* [43]. Similarly, despite demonstrating in vitro activity against *Ascaris suum*, CNM failed to exert any in vivo anthelmintic effect in pigs [44]. In other studies, *C. elegans* exposed to limonene in vitro showed a survival rate of 30% [45]. However, lambs supplemented with orange pulp containing limonene did not exhibit a reduction in either natural or experimental gastrointestinal nematode infections, although the supplementation affected parasite egg hatchability [46].

One of the main pharmacological mechanisms implicated in ML resistance is the upregulation of efflux transporters, particularly P-gps, which limit drug bioavailability at the target site [6,7,8]. The current study builds upon earlier evidence demonstrating that pharmacological inhibition of P-gps can enhance the efficacy of MLs [11,12]. In this context, the combination of DRM with phytochemicals known to interact with P-gp, such as CNM and limonene, offers a rational and innovative strategy [19,20,22]. While CNM–PGF alone exhibits moderate efficacy against resistant gastrointestinal nematodes, its coadministration with DRM significantly improves therapeutic outcomes. The most notable effect was observed against *H. contortus*, where efficacy increased from 0% (DRM alone) to 79% when coadministered with CNM–PGF (Table 2). While no data are currently available regarding P-gp modulation by CNM when coadministered with MLs, limonene has been shown to restore the in vitro efficacy of ivermectin against resistant *H. contortus* and to reduce the expression of P-gp-9 compared to that of worms exposed to ivermectin alone [22]. Long-term administration of limonene through feed, coadministered with a single dose of ivermectin, resulted in an 82% FECR, although the number of adult nematodes remained unaffected [22]. These findings suggest that limonene may reduce worm fecundity and subsequently decrease pasture contamination. In the current study, the combination of DRM and CNM–PGF, which includes limonene, primarily impacted *H contortus*, with no observable effect on *Teladorsagia* spp. or intestinal nematodes. There were some variations in the response to the combination of DRM + CNM–PGF between the two efficacy trials conducted in the present study. Although the main reason for this difference remains unknown, the differential composition of parasite populations may help explain these results. In the first study, *H. contortus*, a genus potentially more susceptible to these phytochemicals, accounted for 48% of the population, whereas in the second study, it represented only 10%.

Intestinal diffusion models are widely employed to evaluate the absorption and transport characteristics of drugs across the intestinal epithelium, particularly for compounds that are substrates of transporter proteins such as P-gp. However, few studies have utilized intestinal tissue from sheep [47]. In the present study, the ER of Rho123 across sheep intestinal tissue was 10.3, confirming that the model effectively detected the active transport of this known P-gp substrate (Figure 1). The ex vivo assessment of intestinal transport provided mechanistic insight into drug–transporter interactions. The presence of CNM–PGF primarily enhanced the absorptive flux of Rho123, as reflected by increased *P_eff_* (M to S) values, without significantly affecting the secretory flux (*P_eff_* S to M). This indicates that CNM–PGF significantly inhibited the efflux of Rho123, in a manner comparable to that of LPM, a well-established P-gp inhibitor [11] (Figure 1), and confirms that phytochemicals may modulate efflux transporters. Interestingly, while ivermectin reduced the ER of Rho123 from 6.49 to 1.12, in the current trial, DRM, another avermectin, reduced the ER from 10.3 to 4.66. This comparatively lower effect observed in the ex vivo model contrasts with findings from in vitro studies, where ivermectin and DRM exhibited similar P-gp inhibitory capacity [23].

Interestingly, despite the marked effects observed in the ex vivo intestinal assay, the in vivo pharmacokinetic profile of DRM was not significantly altered by coadministration with CNM–PGF in the in vivo pharmacokinetic/pharmacodynamic assessment. Plasma concentrations of DRM and the main kinetics parameters such as Cmax, AUC, and elimination half-life, remained comparable across treatment groups (Figure 2, Table 3) in contrast to the modifications observed following the coadministration of ivermectin with carvone [17]. Notably, the coadministration with LPM did not affect the pharmacokinetic disposition of DRM, in contrast to previous findings in which LPM coadministration significantly modified the kinetics of ivermectin in lambs [11]. The use of emulsifying agents in the emulsion preparation, such as Tween 80 and sesame oil, may influence P-gp activity, but they can also introduce confounding effects on the observed impact of the phytochemical under study with respect to drug absorption and transporter function [48,49]. Such excipients may increase DRM absorption, potentially modulating P-gp activity, as previously demonstrated for digoxin in rats [48]. There are limited reports describing the pharmacokinetics of DRM in sheep following oral administration. In a previous study, the absolute oral bioavailability of DRM was reported to be low (25%) [50], which may be attributed to the efflux mediated by P-gp at the intestinal level. In the present trial, higher Cmax and AUC values were observed after oral administration of DRM compared to previously reported values [50]. This enhanced absorption may be related to the formulation used in this study, where DRM was administered as an oral emulsion containing surfactants. Although the coadministration with CNM–PGF and LPM did not alter the systemic pharmacokinetics of DRM, some effects were observed at the parasitic level. In this experimental phase, the primary impact was again observed on *H. contortus* (Table 5). These findings suggest that the enhanced efficacy is more likely attributable to localized drug–drug interactions and increased drug availability at the parasite interface rather than to changes in systemic exposure.

While the current work shows positive results regarding the nematocidal activity of phytochemicals, some limitations must be acknowledged. The relatively low concentrations of CNM and PGF at the parasite level may fall below the thresholds required for full anthelmintic efficacy, suggesting that further optimization of dosage, formulation, or treatment schedule is needed. Additionally, variability in FECRT and differences in parasite genus composition between trials may complicate the interpretation of the results and limit the extrapolation of the findings. Another limitation is the absence of groups of lambs treated with CNM or PGF alone, or each combined separately with DRM, which would have allowed for the identification of the most effective in vivo combination. Recent in vivo administration of different phytochemical combinations to sheep has shown a comparable impact on nematode egg counts in feces, with reductions ranging from 25% to 69% [37,38,39]. These findings highlight the need for further studies to establish the optimal dose, formulation type, and administration schedule of these compounds

## 5. Conclusions

The combination of DRM with CNM–PGF may represent a valuable pharmacological tool to enhance the activity of MLs. Based on the results obtained, the best treatment would be the combination of DRM with phytochemicals such as CNM–PGF and LPM, but with a pharmaceutical formulation that allows a sustained in vivo interaction between these compounds. This study contributes to the growing body of evidence supporting the integration of natural-product-based modulators into antiparasitic therapy, aiming to preserve drug efficacy and delay the development of resistance.

## Figures and Tables

**Table 1:** Mean egg per gram (EPG) counts (±SD) and fecal egg count reduction percentage (*FECR*) obtained 14 days after the oral administration of doramectin (DRM), cinnamaldehyde plus pink grapefruit essential oil (CNM–PGF), and their combination to lambs (*n* = 9) naturally infected with resistant nematodes.

Treatment	Day 0	Day 14	*FECR* (%) (LCL-UCL)
**DRM**	2716 ± 2641 ^aA^	910 ± 2548 ^abB^	66
			(0–98)
**CNM–PGF**	1949 ± 1093 ^aA^	1615 ± 1129 ^aA^	17
			(0–53)
**DRM + CNM–PGF**	2422 ± 1288 ^aA^	529 ± 1587 ^bB^	78
			(34–99)

LCL: lower confidence limit; UCL: upper confidence limit. Different lowercase letters between treatments at different sampling days indicate statistical differences (*p* 0.0021). Different capital letters among sampling days for each treatment indicate statistically different values (*p* 0.0039, *p* 0.0273). Results of the Dunn’s post hoc tests are shown in the Supplementary File.

**Table 2:** Efficacy (%) by nematode genus obtained 14 days after the oral administration of cinnamaldehyde plus pink grapefruit essential oil (CNM–PGF), doramectin (DRM), and their combination to lambs (*n* = 9) naturally infected with resistant nematodes.

	CNM–PGF	DRM	DRM + CNM–PGF
*Haemonchus* spp.	37	0	79
*Teladorsagia* spp.	5.7	100	82
*Trichostrongylus* spp.	0	100	76
*Cooperia* spp.	0	100	100

**Table 3:** Plasma pharmacokinetic parameters of doramectin (DRM) in plasma (mean ± SD) obtained after its oral administration, either alone or coadministered with cinnamaldehyde plus pink grapefruit essential oil (CNM–PGF) or loperamide (LPM), to sheep (*n* = 7).

Kinetic Parameters	DRM	DRM + CNM–PGF	DRM + LPM
Cmax (ng/mL)	25.76 ± 10.01 ^a^	23.73 ± 6.55 ^a^	26.95 ± 13.32 ^a^
T max (days)	0.93 ± 0.47 ^a^	0.80 ± 0.34 ^a^	0.86 ± 0.43 ^a^
AUC_0–t_ (ng d/mL)	90.84 ± 33.61 ^a^	72.03 ± 18.99 ^a^	80.72 ± 31.29 ^a^
T ½ ab (days)	0.26 ± 0.13 ^a^	0.42 ± 0.15 ^a^	0.43 ± 0.33 ^a^
T ½ el (days)	2.19 ± 0.41 ^a^	2.52 ± 0.43 ^a^	2.66 ± 0.43 ^a^

Cmax, peak plasma concentration; Tmax, time to peak plasma concentration; AUC_0–t_, area under concentration vs. time curve from time 0 to the last concentration detected; T ½ ab, absorption half-life; T ½ el, elimination half-life. Different letters between treatments for kinetic parameters indicate statistically different values. The *p* values were >at 0.05 for all parameters.

**Table 4:** Mean egg per gram (EPG) counts (±SD) and fecal egg count reduction percentage (FECR) obtained 14 days after the oral administration of doramectin (DRM), either alone and combined with cinnamaldehyde plus pink grapefruit essential oil (CNM–PGF) or loperamide (LPM), to lambs (*n* = 7) naturally infected with resistant nematodes.

Treatment	Day 0	Day 14	FECR (%) (LCL-UCL)
**DRM**	1586 ± 984 ^aA^	694 ± 944 ^aB^	56
			(9–88)
**DRM + CNM–PGF**	1263 ± 736 ^aA^	762 ± 1565 ^aA^	40
			(0–95)
**DRM + LPM**	1584 ± 782 ^aA^	397 ± 520 ^aB^	75
			(50–92)

LCL: lower confidence limit; UCL: upper confidence limit. Different lowercase letters between treatments at different sampling days indicate statistical differences (*p* 0.8713). Different capital letters among sampling days for each treatment indicate statistically different values (*p* 0.0156). Results of the Dunn’s post hoc tests are shown in the Supplementary File.

**Table 5:** Efficacy (%) by nematode genus obtained 14 days after the oral administration of doramectin (DRM), either alone and combined with cinnamaldehyde plus pink grapefruit essential oil (CNM–PGF) or loperamide (LPM), to lambs (*n* = 7) naturally infected with resistant nematodes.

	DRM	DRM + CNM-GF	DRM + LPM
*Haemonchus* spp.	0	29	79
*Teladorsagia* spp.	82	49	86
*Trichostrongylus* spp.	100	100	95

## Data Availability

The raw data supporting the conclusions of this article will be made available by the authors upon request.

## Supplementary Materials

The following supporting information can be downloaded at: https://www.mdpi.com/article/10.3390/ani15172539/s1, Supplementary data of Table 1 and Table 4.
