# Supplementary material for: Phytochemical-Mediated Modulation of Doramectin Pharmacology in Sheep: Exploring the Cinnamaldehyde–Pink Grapefruit Combination
Source: Animals (Basel). 2025 Aug 29;15(17):2539. doi: 10.3390/ani15172539 (PMC12427341; doi:10.3390/ani15172539)
Supplement: Supplementary file 1 [file animals-15-02539-s001.zip › animals-3835563-supplementary.pdf]

Supplementary data of Table 1

Results of the Dunn's post-hoc tests

Pairwise comparisons	P value	
	Day 0	Day 14
DRM vs CNM-PGF	0.9999	0.0940
DRM vs DRM+CNM-PGF	0.9999	0.5580
DRM+CNM-PGF vs CNM-PGF	0.9999	0.0015

Supplementary data of Table 4

Results of the Dunn's post-hoc tests

Pairwise comparisons	P value	
	Day 0	Day 14
DRM vs DRM+CNM-PGF	0.9999	0.9999
DRM vs DRM+LPM	0.9999	0.9999
DRM+LPM vs DRM+CNM-PGF	0.9999	0.9999
